# Associations of Pet Ownership with Older Adults Eating Patterns and Health

**DOI:** 10.1155/2017/9417350

**Published:** 2017-05-29

**Authors:** Roschelle Heuberger

**Affiliations:** Department of Human Environmental Studies, Central Michigan University, Mt. Pleasant, MI, USA

## Abstract

Pet ownership has been shown to improve quality of life for older adults. The objective of this cross-sectional study was to compare older pet owners and older non-pet owners and assess differences between groups. This study was conducted on adults over 50 years of age, who owned either one cat or one dog versus nonowners based on age, race, gender, and education. Matched older pet owners (OPO) versus non-pet owners (NPO) pairs (*n* = 84), older cat owners (OCO) versus non-cat owners (NCO) (*n* = 29), and older dog owners (ODO) versus non-dog owners (NDO) pairs (*n* = 55) were analyzed. No differences were found between OPO and NPO for dietary, activity, or lifestyle, except OPO had fewer health conditions [*p* < 0.03]. Total OCO had greater body mass indices [BMI] (*μ* = 29.6 ± 8.2) than ODO (*μ* = 23.2 ± 5.2) [*p* < 0.02], less activity [*p* < 0.02], and shorter duration of activity [*p* < 0.05] and took fewer supplements [*p* < 0.003]. OCO and NCO differed on health conditions (*μ* = 0.8 ± 0.9 versus *μ* = 1.9 ± 1.3, [*p* < 0.008]) and ODO versus NDO differed on BMI (*μ* = 25 ± 4 versus *μ* = 27 ± 6, [*p* < 0.04]). Although there are limitations to this study, data may be useful for targeting marketing and health messages to older persons.

## 1. Introduction

Pet ownership is alleged to have beneficial effects on health in older adults [1]; therefore, a study of community dwelling, ill, and debilitated elderly adults was conducted. Pet ownership was evaluated against measures of health in the Netherlands. In a cross-sectional analysis of 12,297 older adults in the Netherlands, 2358 were pet owners. Older adults who owned a dog showed significantly (*p* < 0.001) increased activity and socialization. Older adults who owned a cat showed decreased activity and socialization [2]. In a study of Scandinavians older adults who owned a dog showed overall better health and health related behaviors when compared to older adult non-pet owners and cat owners. Cat ownership was associated with higher blood pressure, worse health status, and less physical activity when compared to older adult non-cat owners (*p* < 0.001) [3].

Dog ownership has been studied and found to increase activity among older adults across all seasons. Authors advocated for policies and programs that encourage walking in geographic areas with harsh seasons using dog friendly parks and neighborhoods and providing support and education to owners [4]. Pet ownership among older adults has also been associated with the use of mental health care, but associations with loneliness or social interactions as a result of having a pet were not found [2]. Human-animal bonds are also a factor but are difficult to measure and can impact quality of life for both the owner and the pet [5]. An “ideal” dog or an educated owner that has realistic expectations of the dog increases owner satisfaction and thus quality of life [6]. Companion animal ownership or interaction has been associated with improving feelings of “wellbeing” among those with illnesses, such as HIV, long term mental illness, congestive heart failure, diabetes, end stage cancer, acute illness, chronic pain, depression, posttraumatic stress disorder, and physical disability [6–18]. However, many studies found clinical benefit, but not necessarily strong statistical significance, possibly due to the complex nature of measuring “wellbeing” and the difficulty in sampling and design in these types of studies.

In recent years, several investigators studied the attachment of people to pets and used “relational” constructs to evaluate the effects of pet ownership and the human-animal bond on overall social satisfaction and healthy aging paradigms. Pet ownership was found to be a positive influence on relationship satisfaction, empathy, social attitude, socialization, and companionship and had postulated direct effects on health, such as increasing serum levels of neurotransmitters and hormones and overriding nociception, attenuating sensory deficits, and decreasing the hemodynamic changes that occur from the stress response [19–30]. In addition, increased ambulation, physical activity (through dog walking), has been found to increase measures of cardiovascular competence, promote health aging, increase one's ability to age in place, and attenuate decrements in performing activities of daily living associated with increasing age [31–36]. Obesity, particularly central adiposity with concomitant loss of muscle and muscle function through infiltration of adipocytes into skeletal and cardiac muscle, has serious implications for morbidity and mortality in older persons. Physical activity through dog walking has also implications for the attenuation of age related sarcopenic obesity, disability, and obesity in general [37–39]. In a cross-sectional study conducted by Utz of 2,474 participants, pet ownership and overall health outcomes were assessed and analyzed. The findings of this study showed that older adults who owned a pet were in overall better health condition. Older adults who owned a pet had less arthritis, healthier weights, and decreased occurrences of congestive heart failure. One of the detriments to owning a pet was that older adults with pets did have increased allergies and asthma. This study emphasizes that pet ownership results in improved overall health [40].

There has also been some data to suggest that dementia patients may benefit from pet assisted therapies, and physical activity, nutrition, agitation, reminiscing, and increased socialization were potentially significant outcomes [41, 42]. In a 2015 study by Freidmann et al., cognitively impaired residents (*n* = 22) were randomized to 60 or 90 minutes of pet assisted therapy and statistically significant improvements were seen in physical, behavioral, and emotional function [43]. Similarly, in a study by Richeson, dementia patients (*n* = 15) who were assigned to an animal assisted therapy protocol showed decrease in agitation and greater social interactions (*p* < 0.001) from baseline [44]. Additionally research has found benefits to persons with dementia with both a robotic and a live dog. This has implications for offsetting the concerns of physical safety, zoonotic infection transmission from animal to human, and damage to property or environment, which is often cited as a rationale for restricting pet therapy in this population [45]. Further research into the cost benefit of pet ownership among older persons is required, but it appears that the benefits may outweigh the risks [46, 47].

The rural United States (US) has a greater proportion of older adults who are impoverished and exhibit greater rates of disease and disability than all other areas of the US. According to the US census, the US Centers for Disease Control and the US National Center for Health Statistics, there are more overweight and obese older persons in parts of the rural US [48]. The National Health Interview data and the Behavioral Risk Factor Surveillance System datasets have shown that a great number of older adults are impaired, are physically inactive, and meet the criteria for disability.

This study was conducted to assess older adults who reside in rural areas of the US, where insufficient descriptive data exist for the relationship between pet ownership and diet, activity, and lifestyle characteristics of the owner as well as the characteristics of their companion animals. The hypotheses included that older dog or cat owners would differ from one another or nonowners with regard to body mass index and select dietary intake variables, number of physician diagnosed diseases, and prescribed medications, related to being an older adult dog owner versus having a cat or being a non-pet owner.

## 2. Materials and Methods

This cross-sectional, unincentivized, convenient investigation was done to evaluate associations of pet ownership to health and weight status of older adult owners. “This study was conducted according to the guidelines laid down in the Declaration of Helsinki and all procedures involving human subjects were approved by the Institutional Review Board and Human Subjects Committee of the primary institution where the research was conducted and informed consent was obtained from all subjects.” In addition, the work which involved analysis of secondary data on animals was approved by the above-mentioned board. All data was rendered anonymous and the use of ID number only in data entry, cleaning, coding, analysis, and dissemination was employed. Data were kept confidential in a secure location and were made available only to authorized researchers at the primary institution granting approval for the study.

### 2.1. Data

Data were not associated with any identifying information and subject confidentiality was maintained. Trained interviewers (*n* = 7), with interrater reliability ratings of Cronbach's alpha = 0.89, solicited pet owner participants from organizations known to be frequented by older adults and pet owners, using flyers, word of mouth, and ads (e.g., Senior Centers, Kiwanis, Red Hats, Pet Care Centers, Clinics, Kennel Clubs, and Guilds). Exclusion criteria consisted of the following: being <18 years of age, being unable to provide informed consent, inability to care for self or cat/dog, having >1 cat or dog/household, refusal to answer >25% of questions, or failure to reside in a rural US locale. Data were split by age >50 years, using this established cut point of the American Association of Retired Persons.

Persons who were not pet owners were continuously recruited until a match was found to a pet owner. Matching was based on age, gender, race, and education. Anyone wanting to participate in the study was allowed to do so, but persons under the age of 50 were excluded from the analyses.

Questionnaires were piloted and focus group input was used to adjust the questions in the questionnaire. Sequential focus group information was used to hone internal validity. Body Condition Scoring Charts (BCS) for pets that had a nine-point scoring system that were available without copyright were used. Scale weight was obtained when available; otherwise owner weights were self-reported, as were the data from non-pet owners. Food intake data was gathered from semiquantitative food frequency questionnaires. Data on exercise was collected using frequency, duration, and intensity scales, with respondent walking for exercise specifically excluding dog walking. Dog walking data was collected in the section devoted to the animal and its care.

### 2.2. Statistical Analysis

Statistical analyses were run using SPSS® v. 23, IBM Corporation, Raleigh-Durham, NC, USA, under license from Central Michigan University, Mount Pleasant, MI 48859. Descriptive statistics, such as frequencies and means, independent sample *t*-tests between matched owner to nonowner pairs, Chi square analysis for older owners and nonowners, and nonparametric statistics for data that were not normally distributed (such as semiquantitative food frequency intake data) were run. Analyses were run on all data with split analyses done by gender. Logistic regression models were run stepwise. Significance was determined by a *p* value of <0.05 for all tests. Trend was determined by a *p* value set at <0.075. Failure to reach statistical significance was denoted by NS.

## 3. Results

### 3.1. Older Owners versus Older Nonowners

**Figure 1:**
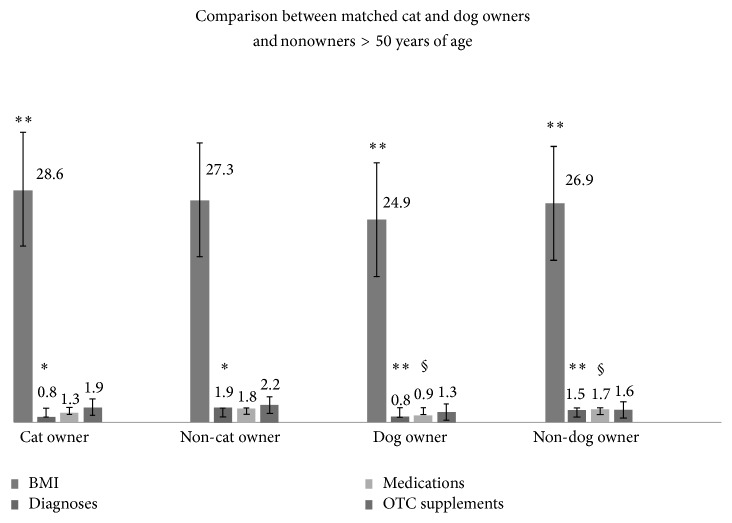
Characteristics of older pet owners versus matched nonowners >50 years residing in the rural United States (*N* = 168). ^*∗*^*p* < 0.01, ^*∗∗*^*p* < 0.05, and ^§^*p* < 0.004.

Split analyses resulted in matched older pet owners (OPO) versus non-pet owners (NPO) usable pairs (*n* = 84), cat owners (OCO) versus non-cat owners (NCO) pairs (*n* = 29), and dog owners (ODO) versus non-dog owners (NDO) pairs (*n* = 55). No significant differences were found between total OPO and NPO for dietary intake, physical activity, or lifestyle characteristics, with exception of OPO having fewer numbers of documented health conditions, despite being matched for demographics using *t*-testing. There were differences between OCO versus NCO and ODO versus NDO on prescribed medication number and BMI (Figure 1). Older pet owners did differ from older non-pet owners on other health related characteristics within groups.

Logistic regressions were run on all age matched owners and nonowners. Regression models showed that the largest contribution to variance in the number of physician diagnosed owner health conditions was pet ownership (Table 1). An increase in BMI was also related to number of owner diagnoses in the sample, and a trend was observed for increased intake of added fat. No other variables, either dietary or lifestyle, contributed significantly to the models.

To investigate contributions by gender, the data were split and analyzed; significant contributions were seen in number of diagnoses for males on BMI, dietary intake of added fats, and servings of whole grains, fruits, and vegetables. In females, pet ownership was found to be significantly related to decrease in disease number, but BMI ceased to be contributory. No other dietary or lifestyle variable was found to contribute significantly among females. It should be noted that there were more females than males in the sample and, thus, data from female respondents' contributed heavily to the findings from the total sample.

### 3.2. Older Dog Owners versus Older Non-Dog Owners

There were 110 ODO and NDO over age of 50 in the sample. Mean age was 56.8 ± 6.4 years; 97% of the sample was Caucasian and 65% female. ODO versus NDO showed significant differences between BMI, number of diagnoses, and prescribed medications using *t*-tests (Figure 1). The dog owners' dogs were, on average, 7.7 ± 4.3 years old and had been owned for *μ* = 7.4 ± 4.3 years. The most commonly owned dog was female (86%) and neutered (100%) and 27% were identified as pure bred Labrador Retriever. Respondents classified their dogs by breed. Respondents were specifically asked if the dog was a mix or pure bred, but no further investigation into lineage was made by interviewers.

In regression models for NDO matched on age to ODO, pet ownership contributed significantly to decreased number of diagnosed conditions in both males and females. In males, increased BMI was significantly related to increased number of health conditions, but the relationship did not hold for females. Smoking and alcohol use did not show statistical significance in the regression models, although a trend was seen between smoking history and increased number of diagnoses in women. In men, dietary intake of whole grain and added fat contributed significantly to model variance (Table 1).

### 3.3. Older Cat Owners versus Older Non-Cat Owners

There were 58 OCO and NCO in this sample. Mean age of the participants was 57.1 ± 6.2 years; 98% were Caucasian and 81% were female. Older CO and NCO differed significantly on number of diagnosed health conditions (*μ* = 0.8 ± 0.9 versus *μ* = 1.9 ± 1.3, [*p* < 0.008]) using *t*-tests. Older cat owners had fewer health problems than NCO despite being matched on available demographics (Figure 1). Their cats were on average 7.3 ± 4.6 years of age and had been owned for *μ* = 6.1 ± 4.8 years. The most commonly owned cat was female (76%), neutered (95%), and shorthaired (32%).

Regression models for diagnosed health conditions among OCO and matched NCO are shown in Table 1. Owning a cat was associated with fewer health problems in the sample, but significance was only seen in females, after the data were split by gender. There were too few males in this sample, which reduced power to detect significance. No other body habitus, lifestyle, or dietary intake variable was contributory in OCO and NCO with respect to number of health conditions in regression models.

### 3.4. Older Dog Owners versus Older Cat Owners

Using nonparametric testing OCO were significantly more likely to be female than ODO (*p* < 0.01). Total OCO had significantly greater body mass indices [BMI = wt.-kg/ht-m^2^] (*μ* = 29.6 ± 8.2) than total ODO (*μ* = 23.2 ± 5.2) [*p* < 0.02], less physical activity [*p* < 0.02], and duration of activity [*p* < 0.05] and took fewer supplements [*p* < 0.003] in *t*-test analyses.

Older pet owners had senior pets, and their senior pets had veterinarian diagnosed health conditions, most commonly allergies (37%) and arthritis (21%) among ODO and allergies (27%) and hyperthyroidism (15%) among OCO. The most frequently used supplement was glucosamine for dogs and a multivitamin for cats. The most common medications were for pain control (22%) in dogs and hyperthyroidism in cats.

### 3.5. Matched Older Cat Owners to Dog Owners to Non-Owners

The most frequently diagnosed health conditions among ODO, OCO, and NPO were allergies followed by hypertension. The over-the-counter supplements used most commonly by OCO, ODO, and NPO were multivitamins, calcium, and fish oil/omega-3 fatty acids, in that order. Walking was the most common form of non-pet-related physical activity reported among NPO, ODO, and OCO. Respondents were specifically asked to separate out walking for exercise without their dog from dog walking. Walking on a treadmill or track, walking in the mall, and walking with a walking group are examples of non-dog walking exercise that was classified as “walking.” To decrease confounding, all three groups were matched for all available demographics yielding 22 usable triads (age *μ* = 55.4 ± 4.5 years). Analyses of these triads revealed no significant differences between NPO, ODO, and OCO using *t*-tests for dietary intake data or lifestyle characteristics. The decreased sample size diminished power to detect differences among groups. Nonowners had slightly but not significantly higher intakes of fruits, vegetables, and whole grains, but lower or equivalent servings of low fat dairy products. Multiple linear regressions for number of owner diagnoses showed significant contributions of pet ownership and BMI, but other variables were NS.

## 4. Discussion

Health and behaviors impacting health can be influenced by pet ownership [49]. An example is increased activity through dog walking [35–37, 50–52]. In this self-selected sample of pet owners >50 years of age matched to non-pet owners on key demographic characteristics, owning a pet was associated with fewer health problems and less prescribed medication. There were differences seen between cat versus dog owners and between those groups and nonowners on variables such as BMI, diagnoses, and health behaviors. The results point to the inherent health benefits of pet ownership for older adults, with dog ownership imparting greater health advantages. This may be due to the increased socialization, tactile stimulation, and psychological deterrent to loneliness that pets provide [53–55]. It has also been shown that companion animals may provide pain relief and stimulate oxytocin production, which increases bonding and feeling needed, which improves quality of life. These indicators are known to influence food consumption, eating patterns, body weight, and body habitus, as well as food choices, meal satisfaction, and appetite. Additionally, there are influences on neurotransmission, chemokines, and inflammation as well as hormones regulating blood pressure [56, 57].

Significant limitations to this study exist, including, but not limited to, convenience sampling, respondent bias, lack of generalizability to other populations, and lack of power to detect significant differences among the matched triads, NPO, ODO, and OCO.

Older adults owning dogs may be an inherently different population than older cat owners; they may be more mobile, active, and predisposed to socialization in the first place. In addition, subjects were only included if they had one pet per household, which is a significant limitation but was necessary to ensure that the data collection on diet and other characteristics were specifically for the one pet in the home. Multiple pets would have presented problems in collecting dietary and activity data, particularly if they were provided food ad libitum. Also, older adults keeping multiple pets in advanced age may be a very different demographic than those with a one pet household. Further research evaluating owning multiple pets among those of advanced age would be beneficial.

Owner demographics, socioeconomics, body habitus, and health are important to consider when advising older clients or marketing to older adults for themselves or their companion animals [58]. Older owners caring for older pets are a research area that should be explored, given the burgeoning older adult population in rural areas of the United States.

## 5. Conclusions

In this sample, rural, older pet owners differed from matched nonowners of companion animals, on several variables, including number of health conditions and BMI. Older cat owners differed from older dog owners, with higher BMIs, less physical activity, and less supplement usage. Older cat owners were much more likely to be female than dog owners and in worse condition. Differences in dietary, lifestyle, or health related characteristics between older cat, dog, and nonowners, when matched to one another on all available demographics, while not statistically significant, showed that pet ownership was indeed beneficial for older persons. Pet ownership and BMI significantly contributed to better overall health, using number of diagnosed conditions as a surrogate marker. Further research in this arena is required, particularly in light of the burgeoning older adult population and the trend towards viewing pets as family members. Older owner lifestyle, health practices, and care decisions may extend to their pet. Treatment options for either the owner or the pet should be tailored in the context of the pet as a family member for enhanced outcomes.

## Figures and Tables

**Table 1:** Regression models of body mass index, dietary, and lifestyle characteristics on health in pet owners and matched nonowners over age of fifty (*N* = 168).

	*All older owners versus matched older nonowners*														
	Total (*N* = 168)					Males (*n* = 50)					Females (*n* = 118)				
	*B*	SE	*β*	*t*	Sig.	*B*	SE	*β*	*t*	Sig.	*B*	SE	*β*	*t*	Sig.

Owns pet	−0.75	0.19	−0.30	−3.9	0.001	−0.73	0.38	−0.27	−1.93	0.06	−0.68	0.23	−0.28	−3.04	0.003
BMI (kg/m^2^)	0.035	0.02	0.16	2.1	0.04	0.08	0.04	0.31	2.17	0.04	0.03	0.02	0.12	1.29	0.20
^‡†^*Model*	^‡^ *R = 0.35; R* ^*2*^ * = 0.12; AR* ^*2*^ * = 0.11; SEE = 1.2*					^‡^ *R = 0.48; R* ^*2*^ * = 0.23; AR* ^*2*^ * = 0.19; SEE = 1.2*					^‡^ *R = 0.34; R* ^*2*^ * = 0.09; AR* ^*2*^ * = 0.08; SEE = 1.2*				
Smoke (pack years)	0.35	0.29	0.20	1.2	0.20	0.44	0.51	0.33	0.86	0.40	0.91	0.33	0.14	0.58	0.60
Alcohol (drink/week)	0.20	0.25	0.15	0.8	0.40	0.51	0.55	0.36	0.95	0.40	0.30	0.39	0.18	0.78	0.40
^†^*Model*	*R = 0.28; R* ^*2*^ * = 0.08; AR* ^*2*^ * = 0.01; SEE = 1.5*					*R = 0.42; R* ^*2*^ * = 0.18; AR* ^*2*^ * = −0.09; SEE = 1.6*					*R = 0.26; R* ^*2*^ * = 0.07; AR* ^*2*^ * = −0.03; SEE = 1.54*				
Fruit, vegetable (serving/week)	−0.01	0.01	−0.06	−0.07	0.50	−0.02	0.02	−0.19	−1.13	0.30	0.001	0.01	0.001	0.001	1.00
Whole grain (serving/week)	0.02	0.02	0.12	1.4	0.20	0.05	0.02	0.39	2.42	0.02	0.01	0.02	0.06	0.55	0.60
Low fat dairy (serving/week)	−0.01	0.01	−0.06	−0.60	0.50	−0.04	0.02	−0.23	−1.50	0.10	−0.01	0.02	−0.04	−0.35	0.70
Fast food (serving/week)	0.04	0.15	0.03	0.30	0.80	−0.09	0.23	−0.06	−0.40	0.70	0.05	0.19	0.03	0.28	0.80
Added fat (serving/week)	0.20	0.11	0.16	1.83	0.07	0.58	0.19	0.44	3.13	0.003	0.001	0.13	0.001	−0.01	0.90
Fish (serving/week)	−0.03	0.13	−0.02	−0.21	0.80	−0.35	0.25	−0.20	−1.40	0.20	0.07	0.15	0.05	0.48	0.60
^†^*Model*	*R = 0.21; R* ^*2*^ * = 0.04; AR* ^*2*^ * = 0.01; SEE = 1.3*					^‡^ *R = 0.62; R* ^*2*^ * = 0.39; AR* ^*2*^ * = 0.28; SEE = 1.2*					*R = 0.08; R* ^*2*^ * = 0.01; AR* ^*2*^ * = −0.06; SEE = 1.3*				

	*Older dog owners versus matched non-dog owners*														
	Total (*N* = 110)					Males (*n* = 40)					Females (*n* = 70)				
	*B*	SE	*β*	*t*	Sig.	*B*	SE	*β*	*t*	Sig.	*B*	SE	*β*	*t*	Sig.

Owns pet	−0.67	0.23	−0.27	−2.95	0.004	−0.71	0.37	−0.30	−1.94	0.06	−0.64	0.29	−0.26	−2.21	0.03
BMI (kg/m^2^)	0.06	0.03	0.22	2.27	0.03	0.13	0.04	0.51	3.48	0.001	0.06	0.04	0.2	1.7	0.10
^†^*Model*	^‡^ *R = 0.32; R* ^*2*^ * = 0.10; AR* ^*2*^ * = 0.09; SEE = 1.2*					^‡^ *R = 0.30; R* ^*2*^ * = 0.09; AR* ^*2*^ * = 0.07; SEE = 1.2*					^‡^ *R = 0.26; R* ^*2*^ * = 0.07; AR* ^*2*^ * = 0.05; SEE = 1.2*				
Smoke (pack years)	0.89	0.42	0.48	2.1	0.06	0.19	0.26	0.46	0.72	0.55	1.3	0.60	0.59	2.17	0.06
Alcohol (drink/week)	0.38	0.34	0.25	1.1	0.30	0.13	0.34	0.23	0.37	0.75	0.34	0.41	0.22	0.82	0.44
^†^*Model*	*R = 0.58; R* ^*2*^ * = 0.33; AR* ^*2*^ * = 0.23; SEE = 1.4*					*R = 0.47; R* ^*2*^ * = 0.22; AR* ^*2*^ * = −0.56; SEE = 0.56*					*R = 0.69; R* ^*2*^ * = 0.47; AR* ^*2*^ * = 0.34; SEE = 1.50*				
Fruit, vegetable (serving/week)	−0.01	0.01	−0.12	−0.97	0.30	−0.03	0.02	−0.28	−1.40	0.17	−0.01	0.02	−0.04	−0.27	0.79
Whole grain (serving/week)	0.02	0.01	0.19	1.69	0.10	0.05	0.02	0.46	2.38	0.03	0.01	0.02	0.09	0.63	0.53
Low fat dairy (serving/week)	−0.01	0.02	−0.06	−0.48	0.60	−0.03	0.03	−0.25	−1.36	0.18	−0.001	0.02	−0.01	−0.06	0.95
Fast food (serving/week)	0.03	0.17	0.02	0.15	0.90	−0.13	0.24	−0.09	−0.53	0.60	0.036	0.25	0.02	0.15	0.89
Added fat (serving/week)	0.32	0.14	0.24	2.3	0.02	0.51	0.20	0.43	2.52	0.02	0.16	0.19	0.12	0.87	0.39
Fish (serving/week)	−0.02	0.15	−0.02	−0.16	0.87	−0.23	0.26	−0.14	−0.86	0.40	0.02	0.20	0.02	0.11	0.91
^‡†^*Model*	^‡^ *R = 0.31; R* ^*2*^ * = 0.09; AR* ^*2*^ * = 0.03; SEE = 1.3*					^‡^ *R = 0.63; R* ^*2*^ * = 0.40; AR* ^*2*^ * = 0.26; SEE = 1.09*					*R = 0.14; R* ^*2*^ * = 0.02; AR* ^*2*^ * = −0.09; SEE = 1.32*				

	*Older cat owners versus matched non-cat owners*														
	Total (*N* = 58)					Males (*n* = 12)					Females (*n* = 46)				
	*B*	SE	*β*	*t*	Sig.	*B*	SE	*β*	*t*	Sig.	*B*	SE	*β*	*t*	Sig.

Owns pet	−1.2	0.30	−0.43	−3.57	0.001	−1.37	0.89	−0.45	−1.53	0.16	−0.99	0.316	−0.43	−3.15	0.003
BMI (kg/m^2^)	0.013	0.03	0.07	0.50	0.62	0.04	0.09	0.14	0.40	0.70	0.01	0.03	0.05	0.33	0.74
^‡†^*Model*	^‡^ *R = 0.43; R* ^*2*^ * = 0.19; AR* ^*2*^ * = 0.17; SEE = 1.1*					*R = 0.45; R* ^*2*^ * = 0.21; AR* ^*2*^ * = 0.12; SEE = 1.48*					^‡^ *R = 0.43; R* ^*2*^ * = 0.18; AR* ^*2*^ * = 0.16; SEE = 1.08*				
Smoke (pack years)	−0.07	0.39	−0.06	−0.19	0.85	0.60	1.72	0.36	0.35	0.79	−0.24	0.42	−0.21	−0.58	0.58
Alcohol (drink/week)	0.02	0.34	0.01	0.04	0.97	0.20	1.4	0.15	0.14	0.91	−0.17	0.44	−0.14	−0.38	0.71
^†^*Model*	*R = 0.06; R* ^*2*^ * = 0.003; AR* ^*2*^ * = −0.16; SEE = 1.5*					*R = 0.33; R* ^*2*^ * = 0.11; AR* ^*2*^ * = −1.67; SEE = 3.13*					*R = 0.29; R* ^*2*^ * = 0.09; AR* ^*2*^ * = −0.14; SEE = 1.27*				
Fruit, vegetable (serving/week)	−0.004	0.02	−0.05	−0.23	0.82	−0.01	0.07	−0.11	−0.22	0.84	0.00	0.02	0.01	0.02	0.98
Whole grain (serving/week)	0.01	0.03	0.08	0.46	0.65	0.95	0.11	0.35	0.84	0.47	0.02	0.03	0.12	0.62	0.54
Low fat dairy (serving/week)	−0.01	0.03	−0.07	−0.43	0.67	0.14	0.15	0.41	0.93	0.42	−0.01	0.03	−0.07	−0.35	0.73
Fast food (serving/week)	0.01	0.3	0.01	0.03	0.97	−0.10	0.86	−0.04	−0.11	0.92	0.04	0.35	0.02	0.11	0.92
Added fat (serving/week)	0.03	0.21	0.02	0.14	0.89	1.20	0.55	0.75	2.17	0.12	−0.24	0.22	−0.20	−1.10	0.28
Fish (serving/week)	0.06	0.30	0.04	0.20	0.85	−0.86	1.04	−0.37	−0.82	0.47	0.25	0.30	0.16	0.83	0.41
^†^*Model*	*R = 0.11; R* ^*2*^ * = 0.01; AR* ^*2*^ * = −0.14; SEE = 1.39*					*R = 0.84; R* ^*2*^ * = 0.70; AR* ^*2*^ * = 0.10; SEE = 1.49*					*R = 0.25; R* ^*2*^ * = 0.06; AR* ^*2*^ * = −0.13; SEE = 1.31*				

^†^Constant. ^‡^Statistically significant at the level of *p* < 0.05.

## Additional Points

(i) Older adult pet owners have overall better health with regard to weight, health conditions, and fewer medications. (ii) Older adult dog owners have better health status than older adult cat owners. (iii) Pets help keep older adults more physically active and decrease loneliness and physical and cognitive decline, which improves quality of life and overall health.
